# Unveiling the Genomic Landscape of Yan Goose (*Anser cygnoides*): Insights into Population History and Selection Signatures for Growth and Adaptation

**DOI:** 10.3390/ani16020194

**Published:** 2026-01-08

**Authors:** Shangzong Qi, Zhenkang Ai, Yuchun Cai, Yang Zhang, Wenming Zhao, Guohong Chen

**Affiliations:** 1 Key Laboratory for Evaluation and Utilization of Poultry Genetic Resources, Ministry of Agriculture and Rural Affairs, Yangzhou University, Yangzhou 225009, China; mz120221524@stu.yzu.edu.cn (S.Q.); azk3615@163.com (Z.A.); mx120250902@stu.yzu.edu.cn (Y.C.); wmzhao@yzu.edu.cn (W.Z.); ghchen2019@yzu.edu.cn (G.C.); 2 Key Laboratory for Evaluation and Utilization of Livestock and Poultry Resources (Poultry), Ministry of Agriculture and Rural Affairs, Yangzhou 225009, China; 3 Joint International Research Laboratory of Agriculture and Agri-Product Safety, The Ministry of Education of China, Yangzhou University, Yangzhou 225009, China

**Keywords:** Yan goose, whole-genome resequencing, genetic structure, IHS selection signals, germplasm traits

## Abstract

The Yan goose (YE, *Anser cygnoides*) is a valuable indigenous poultry genetic resource, renowned for its superior meat quality and environmental adaptability. Despite its economic importance, the genetic basis underlying these adaptive traits remains unclear. In this study, we employed whole-genome resequencing (WGS) to perform high-throughput sequencing on a conserved population of 15 samples. Bioinformatic analyses were conducted to systematically evaluate the population’s genetic structure, and a genome-wide scan for selection signals related to economically significant traits was performed using the integrated haplotype score (iHS) method. An average of 4.43 million high-quality SNPs were identified, which were predominantly located in intergenic and intronic regions. Population structure analysis revealed a close genetic relationship within the conserved population of YE, with no significant lineage stratification observed. Pairwise sequentially Markovian coalescent (PSMC) analysis indicated that the YE underwent a severe genetic bottleneck during the Last Glacial Maximum (LGM), followed by gradual population recovery in the early Neolithic period. Genome-wide selection signal scanning identified multiple genomic regions under strong selection, annotating key genes associated with growth and development (e.g., *GHRL*, *AKT1*, and *MAPK3*), lipid deposition (e.g., *PLPP4*, *SAMD8*, and *LPIN1*), and disease resistance and stress resilience (e.g., *TP53*, *STAT3*). Functional enrichment analysis revealed significant enrichment of these genes in pathways related to glycerophospholipid metabolism (*p* < 0.01), purine metabolism (*p* < 0.01), and immune response (*p* < 0.01). This study not only provides a theoretical foundation for the scientific conservation of the YE germplasm resources but also offers valuable genomic resources for identifying functional genes underlying important economic traits and advancing molecular breeding strategies.

## Simple Summary

The Yan goose (*Anser cygnoides*) is a valuable indigenous poultry breed in China, known for its large body size, high-quality meat, and adaptability to various environments. Despite its economic importance, the genetic basis of these traits remains poorly understood. In this study, we performed whole-genome resequencing on a conserved population of Yan geese to assess their genetic diversity and evolutionary history. Our analysis reconstructed the population’s demographic trajectory, highlighting its resilience to past climatic changes. We also identified key genomic regions and candidate genes associated with growth, lipid metabolism, and immune response, which are crucial for the species’ commercial value. These findings enhance our understanding of the molecular mechanisms underlying the Yan goose’s unique characteristics and provide valuable genomic resources that can support its conservation and future breeding programs aimed at improving poultry industry traits.

## 1. Introduction

The domestic goose, as one of the earliest domesticated poultry species, has a domestication history tracing back to 7000 years ago in eastern China [1]. Through natural environmental adaptation and artificial selection, the domestic goose has gradually developed a rich variety of local breeds. These breeds not only exhibit distinct characteristics in terms of body morphology and production performance but also harbor unique genetic resources and adaptive traits. As one of the countries with the richest goose genetic resources in the world, China currently includes 31 goose breeds in the “National Directory of Animal and Poultry Genetic Resources” (2021 edition), with 30 local breeds and one improved breed [2]. The Yan goose (YE, *Anser cygnoides*), known for its unique morphological features, excellent meat performance, and strong environmental adaptability, is a typical representative of the gray domestic goose breeds in China [3]. Specifically, the body weight of Yan geese can reach 3.5 to 4.0 kg at 70 days of age, which is significantly higher than that of other indigenous breeds such as the Wuzong goose (2.8 kg) and Baizi goose (2.6 kg), classifying it as a medium-sized meat-type breed [4]. Due to its unique germplasm characteristics and economic value, YE was included in the “National Directory of Livestock and Poultry Genetic Resources” in 2000 and was reconfirmed in 2006, becoming one of the key protected livestock and poultry genetic resources. However, with the promotion of modern intensive farming practices and the introduction of foreign breeds, many local goose breeds, including YE, face challenges such as declining population numbers, loss of genetic diversity, and increased inbreeding risks [5]. Therefore, there is an urgent need to strengthen the protection of genomic information for these species.

Genetic diversity is the cornerstone of a species’ evolutionary potential and adaptive capacity, and it is also at the core of genetic resource conservation and sustainable utilization [6]. Traditional genetic assessments often rely on pedigree records, microsatellite markers, or mitochondrial DNA fragment analysis. While these methods have provided insights into maternal lineage origins and genetic structure to some extent, their ability to resolve lineage information is limited, making it difficult to capture genomic-level variations comprehensively [7]. Wei et al. investigated the genetic diversity of the Sanzhou-Black goose and local goose breeds from *Guangdong Province* using mitochondrial DNA, specifically the *Cytochrome C Oxidase Subunit I* gene and the *Displacement Loop* region. While these studies revealed maternal genetic diversity in the extranuclear genome, they were unable to reflect the overall genetic background and selection history of the nuclear genome [8]. With the advancement of high-throughput sequencing technology, WGS has become a powerful tool for analyzing genetic diversity, population structure, and adaptive evolution of species. One such tool is the iHS method, a widely used statistical approach for detecting recent positive selection [9]. The iHS identifies genomic regions under strong selection in populations by comparing the extended homozygosity of ancestral versus derived alleles around core SNP sites and calculating a standardized statistic [10]. The use of selection signal detection methods to study the evolutionary and domestication history of populations plays a crucial role in uncovering potential regions under selection for important economic traits and their genetic mechanisms. This method has been successfully applied in genetic research of multiple livestock and poultry species. It has been widely used to identify candidate genes associated with economic traits in pigs [11], sheep [12], and chickens [13]. Despite significant progress in whole-genome resequencing and selection signal analysis for many local goose breeds, systematic genomic studies on YE, an important local genetic resource, remain limited.

This study aims to utilize whole-genome resequencing technology to perform high-throughput SNP detection on the conserved population of YE. By integrating genetic diversity analysis, population structure assessment, and iHS selection signal scanning, we will comprehensively reveal its genetic characteristics and germplasm potential. This research will not only provide a scientific basis for the conservation and utilization of YE germplasm resources but also offer crucial data support for future molecular breeding and functional gene discovery in geese.

## 2. Materials and Methods

### 2.1. Ethics Approval

All animal experiments were approved by the Institutional Animal Care and Use Committee of the Yangzhou University (Approval Number: 202203621). All procedures were performed in accordance with the Regulations on the Management of Laboratory Animal Affairs (Yangzhou University, 2012) and the Standards for the Management of Experimental Practices (Jiangsu, China, 2008).

### 2.2. Experimental Design and Facilities

**Figure 1:**
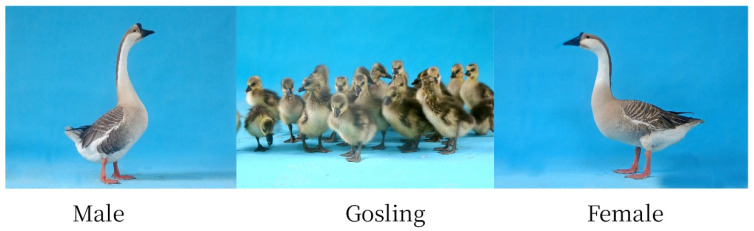
Photographs of individual Yan geese from the conservation population.

This study selected the YE from the National Waterfowl Gene Bank (off-site conservation facility, Taizhou, China) as the research subject (Figure 1). The population is maintained as a closed flock for conservation breeding; however, a rotational mating system is implemented to minimize inbreeding and preserve genetic diversity. For sample collection, we utilized pedigree records from the conservation farm to guide the selection process. Individuals were randomly selected from distinct, unrelated families within the National Waterfowl Gene Bank to maximize the representation of the breed’s genetic diversity and minimize biases arising from kinship. After restraining the geese and disinfecting their wings, venous blood samples were collected from 15 male geese at 70 days of age (15♂) using a venipuncture method. The blood samples were stored in tubes containing anticoagulants and kept at −20 °C until DNA extraction.

### 2.3. Genomic DNA Extraction and Library Construction

Genomic DNA Extraction and Sequencing Genomic DNA was isolated using the TIANamp Genomic DNA Kit (YDP304-03, TIANGEN Biotech, Beijing, China), strictly adhering to the manufacturer’s protocol. The integrity of the DNA was assayed via 1% agarose gel electrophoresis to preclude degradation and contamination. Purity was verified using a UV spectrophotometer to ensure an A260/280 absorbance ratio of 1.8–2.0. DNA concentration was quantified using a Qubit^®^ 2.0 fluorometer (Life Technologies, Carlsbad, CA, USA), yielding a mean concentration of 227 ± 58.2 ng/µL. Library Construction and Bioinformatic Analysis. Upon obtaining high-quality genomic DNA, samples were dispatched to Jisihuiyuan Biotechnology Co., Ltd. (Nanjing, China) for library construction. Sequencing was executed on the Illumina NovaSeq 6000 platform (Illumina, San Diego, CA, USA), employing a paired-end 150 bp (PE150) strategy with an insert size of approximately 350 bp and an average sequencing depth of 6×. Subsequent to quality control of the raw reads, sequences were mapped to the reference genome using the BWA-MEM algorithm. The reference genome of the goose breed was sourced from the National Center for Biotechnology Information database, and the website is (https://www.ncbi.nlm.nih.gov/datasets/genome/GCF_002166845.1/, accessed on 5 January 2026). The SNP calling was implemented via the Genome Analysis Toolkit [14]. Following rigorous filtering, the resultant SNP markers were utilized for haplotype phasing and integrated haplotype score analysis.

### 2.4. SNP Detection and Annotation

Single-nucleotide polymorphism detection was primarily conducted using the GATK v4.1 Based on the alignment results of the clean reads to the reference genome, GATK was utilized to identify SNPs and obtain the final SNP set for subsequent statistical analyses [15]. The core detection process encompassed the following steps: (1) PCR duplicates were removed from the BWA-aligned reads using Picard’s Mark Duplicates tool to mitigate their influence. (2) Variant calling, encompassing both SNPs and Indels, was performed using GATK. (3) Variant quality scores were recalibrated using GATK’s Variant Quality Score Recalibration procedure. (4) The resulting variants were subjected to rigorous filtering using GATK to retain only reliable polymorphisms. Following the initial mapping, a Bayesian approach was implemented for SNP calling in each group. Individual SNPs were detected using the “mpileup” command. To mitigate errors in SNP calling caused by misalignment and Indels, only high-quality SNPs were retained based on the following criteria: minimum coverage depth (≥4) and maximum coverage depth (≤200), root mean square (RMS) mapping quality (≥20), minimum distance between adjacent SNPs (≥5 bp), absence of an Indel within a 3 bp window, and a maximum missing rate of 50% within each group. These retained variants were subsequently annotated, and their effects predicted using SnpEff v5.0. Variant Call Format (VCF) files, compatible with community tools (https://github.com/vcftools/vcftools, accessed on 5 January 2026), served as both input and output for this process.

### 2.5. Characterization of Population Genetic Structure

Following the filtration process, population statistics, including SNP counts, identity-by-state (IBS) genetic distances, inbreeding coefficients (F), and linkage disequilibrium (LD), were calculated utilizing the populations module within the Stacks software suite (v2.59). The estimation of Runs of Homozygosity based on autosomal SNPs was conducted for each individual employing PLINK (v1.9). Principal component analysis (PCA) was performed using the GCTA software (v1.92.1). LD analysis was executed using PopLDdecay (https://github.com/BGI-shenzhen/PopLDdecay, accessed on 5 January 2026) with the following specific parameters: -MaxDist 500, -Het 0.1, -Miss 0.3, and -OutPairLD 5. The chromosomal distribution of LD was visualized via LD decay plots, which characterize the rate of LD decay relative to physical or genetic distance [16]. To reconstruct the demographic history, the PSMC model (v0.6.5-r67) was employed to infer historical fluctuations in effective population size (Ne) based on genomic fragments exhibiting varying densities of heterozygous sites. Furthermore, a maximum likelihood population tree was constructed using the available genetic data to delineate migration events across the phylogenetic topology (specifically for scenarios involving four or more populations).

### 2.6. Detection of Selection Signatures Using the iHS Method

To elucidate potential positive selection regions across the YE genome, selection signatures were assessed using the iHS method [9]. The analysis utilized SNP data generated from the whole-genome resequencing efforts. Initially, the raw SNP data underwent stringent quality control (QC). To ensure the accuracy and robustness of subsequent analyses [17], loci were filtered out if they exhibited a minor allele frequency (MAF) less than 0.05, a missing rate exceeding 10%, or a significant deviation from Hardy–Weinberg equilibrium (HWE; *p* < 1 × 10^−6^). The filtered high-quality data were then subjected to haplotype phase inference using Beagle v5.4 software. This inference was informed by homologous sequence alignment results from the ancestral Wild Swan goose (*Anser cygnoides*). Upon obtaining the phased haplotypes, the extended haplotype homozygosity (EHH) was calculated for the haplotype set carrying both the ancestral and derived alleles, with each SNP locus serving as the core reference point. Subsequently, the EHH values were integrated over distance [18] to derive the integrated haplotype homozygosity (iHH) for each respective allele, calculated according to the following formula:

$${iHH}^{C}\left(s\right)\;=\int_{0}^{d_{max}}{EHH}_{C}(x)dx$$

Here, EHH*_C_* denotes either the ancestral or derived allele. The integration proceeds until the EHH value decays to a threshold of 0.05 or reaches the chromosomal terminus. Based on the computed iHH values for both allelic states, the unstandardized iHS statistic (uniHS) is derived as follows:

$$uniHS(s)=ln(\frac{{iHH}^{A}(s)}{{iHH}^{D}(s)})$$

Given the intrinsic dependency of iHS values on allele frequencies, a standardization procedure was implemented to mitigate this bias. Specifically, all identified loci were stratified into 20 bins based on their derived allele frequencies (DAF). Within each frequency bin, the uniHS scores were normalized to yield the final standardized iHS statistic, calculated as follows:

$${iHS}_{std}(s)=\frac{uniHS(s)-\mu_{bin}}{\sigma_{bin}}$$

where $\mu_{bin}$ and $\sigma_{bin}$ denote the mean and standard deviation, respectively, of the uniHS values within the corresponding frequency bin. To visualize and validate the selection signatures, the distribution of iHS scores was examined, and Manhattan plots were constructed employing the rehh v3.2.1 within the R-statistical environment. Genomic regions exhibiting elevated selection signals were subsequently annotated and subjected to functional enrichment analysis. Gene Ontology (GO) functional annotation and Kyoto Encyclopedia of Genes and Genomes (KEGG) pathway enrichment analyses were conducted for the candidate genes utilizing the metascape database [19]. Significantly enriched GO terms and KEGG pathways were identified based on a Benjamini–Hochberg-corrected *p*-value threshold.

## 3. Results

### 3.1. Quality Control and Assessment

#### 3.1.1. Quality Control and Genome Alignment

In this study, we conducted whole-genome resequencing of the local goose population to characterize its genomic variations. The sequencing data exhibited high integrity and stability following rigorous quality control protocols (Table S1). The average clean data yield per sample was 7.02 Gb, with mean Q20 and Q30 scores of 97.33% and 91.04%, respectively. The GC content was uniform across samples, averaging 43.38%. Subsequent alignment to the reference genome demonstrated excellent compatibility (Table S2), achieving a mean alignment rate of 98.30% and an average sequencing depth of 6.43×. These metrics confirm that the data quality satisfies the requirements for reliable genome-wide SNP detection and population genetic analyses.

#### 3.1.2. Identification and Annotation of SNP Variants

Genome-wide SNP variant detection was performed on the sample cohort (Table 1). The results revealed discernible variation in SNP abundance across individuals. The mean SNP count stood at 4,429,082, ranging from 4,249,848 (YE.1) to 4,868,607 (YE.5). Regarding variation types, both transitions (Ti) and transversions (Tv) were identified. The transition-to-transversion ratio (Ti/Tv) remained consistent across samples, ranging between 2.45 and 2.49 with a mean value of 2.47. This ratio conforms to the canonical patterns observed in most vertebrate species, attesting to the biological validity of the identified variants and their consistency with established genomic norms. In terms of zygosity, the average counts of heterozygous and homozygous SNPs were 2,185,710 and 2,243,372, respectively. The slight preponderance of homozygous variants over heterozygous ones aligns with the characteristic genomic stability of the YE. Notably, sample YE.5 exhibited a distinctly elevated heterozygosity count (2,517,574), potentially indicative of greater individual genetic diversity, whereas sample YE.6 displayed the lowest count (1,825,696).

#### 3.1.3. Genomic Distribution and Functional Annotation of SNPs

Subsequent analyses were conducted based on the statistical distribution of SNP annotations across the sample cohort (Table 2). The distribution of SNPs across all samples was predominantly concentrated within intergenic regions. With a mean count of 1,167,101, these variants constituted a substantial proportion of the total SNP dataset, significantly exceeding the counts observed in other functional genomic elements. Regarding functional coding regions, the average counts of synonymous and non-synonymous mutations in exons were 33,677 and 11,735, respectively. The prevalence of synonymous mutations suggests that the majority of coding variants are likely neutral, potentially exerting a minimal impact on phenotypic expression. Conversely, intronic regions exhibited a consistently high frequency of variants, with a mean of 1,742,040 SNPs. This observation highlights the substantial genetic diversity harbored within the intronic landscape of the YE genome, which may hold implications for alternative splicing regulation. In the proximal regulatory regions, the average SNP counts for upstream and downstream regions were 62,680 and 68,101, respectively, indicating a considerable degree of variation within these gene regulatory elements. Notably, sample YE.5 displayed a significantly elevated upstream SNP count (77,569) compared to other individuals, potentially correlating with enhanced diversity within its gene regulatory sequences. In contrast, sample YE.1 presented a lower upstream SNP count of 57,391, which may imply relative stability in its gene expression regulatory mechanisms.

### 3.2. Genetic Structure Analysis of Population

#### 3.2.1. Population Structure and Demographic History

**Figure 2:**
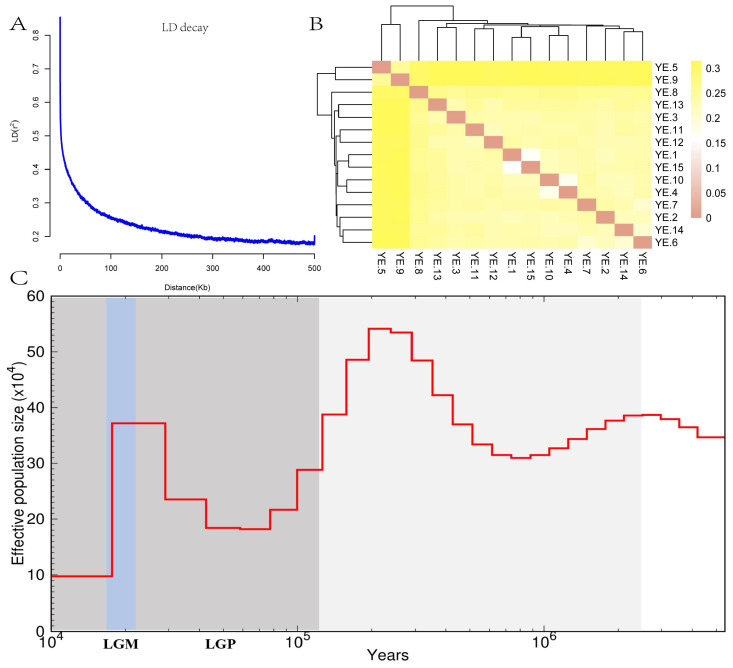
Population genetic structure and demographic history: (**A**) Analysis of linkage disequilibrium decay. The plot illustrates the rate of LD decay (r^2^) as a function of physical distance (kb) within the YE goose. (**B**) Visualization of the intra-population genomic relationship matrix. The heatmap depicts the genetic relatedness among individuals. (**C**) Inference of historical effective population size fluctuations using the PSMC model. The trajectory represents the dynamic changes in inferred Ne over time. The solid line denotes the estimated population size. Background shading indicates specific geological epochs: the grey region represents the Pleistocene era; the dark grey band corresponds to the Last Glacial Period; and the light blue area marks the Last Glacial Maximum.

To characterize the genetic diversity and population structure of the protected YE, we conducted IBD distance analysis, LD decay analysis, G-matrix visualization, and inference of Ne fluctuations using the PSMC model (Table 3 and Figure 2). The results indicate that the mean IBD genetic distance across the population was predominantly distributed between 0.22 and 0.26, suggesting a relatively close genetic relationship at the overall population level. However, samples YE.5 and YE.9 exhibited an elevated IBD distance of 0.31 relative to all other individuals, a value substantially exceeding the population mean. This suggests a potential origin from a relatively distinct or independent genetic background. Conversely, individuals such as YE.1, YE.2, YE.3, and YE.4 displayed lower inter-individual genetic distances, ranging from 0.22 to 0.24, indicating the formation of a closely related kinship unit within the population.

The LD decay curve for the YE exhibited a rapid decline, with the **r**^2^ value dropping below 0.3 at approximately 50 kb (Figure 2A). This observation is indicative of a low level of linkage disequilibrium within the population and a high frequency of recombination events. Consequently, this suggests a high degree of genetic diversity and sufficient gene flow among populations, implying that a relatively random mating scheme has been maintained during the artificial rearing and conservation process. Moreover, the visualization of the G-matrix (Figure 2B) revealed a largely uniform genetic relationship among the majority of individuals in the YE. Only a limited number of pairs exhibited moderate correlation, with no evidence of clear genetic differentiation or pronounced kinship clustering being observed. The PSMC model inference (Figure 2C) demonstrated that the Ne of the YE has undergone periodic fluctuations, characterized by two major bottleneck events. Following the Last Glacial Period (LGP, 2.3 × 10^4^~1.1 × 10^5^ years ago), a relatively warm geological epoch, Ne initially showed a sustained decrease before gradually rebounding. However, Ne experienced a sharp decline to its minimum during the Last Glacial Maximum (LGM, 1.5~2.1 × 10^4^ years ago), a period of severe cold, and subsequently stabilized to a plateau around 10 thousand years ago. This demographic history is potentially attributable to a combination of factors, including artificial domestication, population migration events, or increased selection pressure during modern breeding practices.

#### 3.2.2. Assessment of Genomic Inbreeding and Heterozygosity

**Figure 3:**
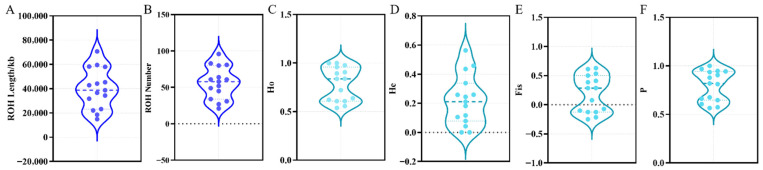
Genomic diversity and inbreeding assessment of the Yan goose population: (**A**) Distribution of total Runs of Homozygosity (ROH) length (kb) per individual. (**B**) Distribution of the total number of ROH segments per individual. (**C**) Observed heterozygosity (Ho). (**D**) Expected heterozygosity (He). (**E**) Genomic inbreeding coefficient (Fis). (**F**) Polymorphism information content (P). The violin plots illustrate the data density distribution, while the dots represent individual samples. The dashed lines within the violin plots indicate the median and quartiles.

To further provide a quantitative assessment of the population’s genetic diversity and inbreeding levels, we analyzed Runs of Homozygosity (ROH), heterozygosity, and inbreeding coefficients (Figure 3). The ROH analysis (Figure 3A,B) showed that the genomic burden of homozygosity is moderate, with the total ROH length per individual varying within a reasonable range. The observed heterozygosity (Ho) and expected heterozygosity (He) distributions (Figure 3C,D) indicated a balanced genetic variability within the population. Crucially, the genomic inbreeding coefficient (Fis) for most individuals was centered around zero (Figure 3E), confirming that the population does not suffer from severe inbreeding depression. These metrics collectively suggest that the current conservation strategy, utilizing a rotational mating system, has been effective in maintaining the genetic diversity of the YE.

### 3.3. Detection of Selection Signatures

#### 3.3.1. Population-Level Selection Signal Detection

**Figure 4:**
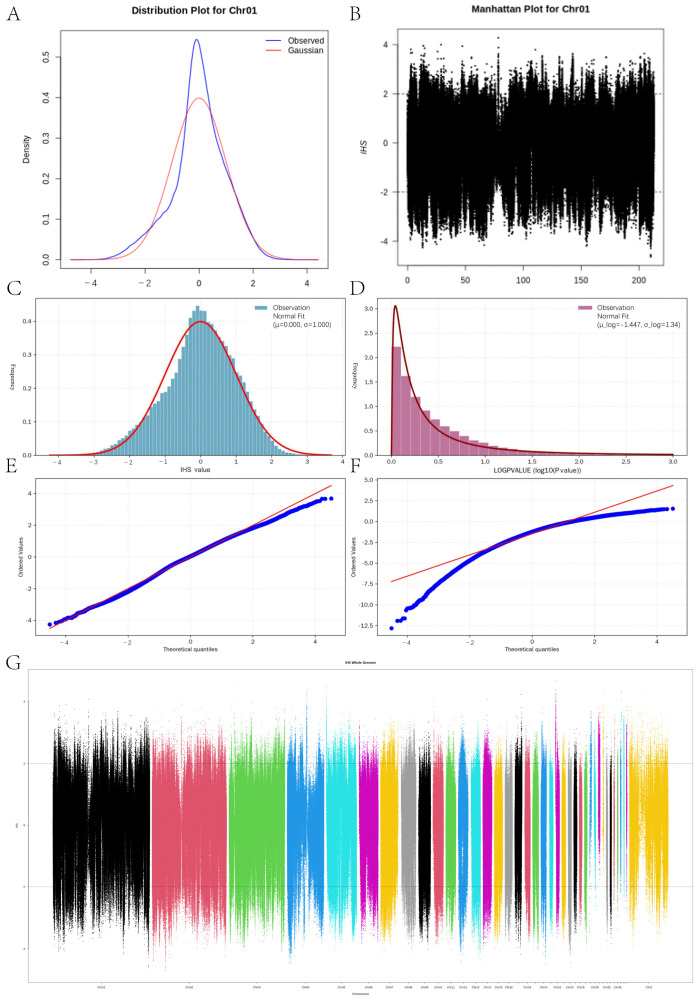
Distributional characteristics of genome-wide selection statistics: (**A**) Selection signal distribution on the YE Chromosome 1 (Chr 01). (**B**) Manhattan plot of selection signal distribution on Chr 01. (**C**) Frequency distribution histogram of standardized iHS values. The blue bars represent the observed frequency of iHS scores across the entire genome, while the red curve denotes the fitted standard normal distribution. (**D**) Frequency distribution histogram of LOGPVALUE. The pink bars illustrate the pronounced right-skewed distribution. (**E**) Quantile–quantile (Q-Q) plot of iHS values. (**F**) Quantile–quantile (Q-Q) plot of LOGPVALUE. (**G**) Genome-wide Manhattan plot of iHS values. And different colors represent different chromosome positions.

To elucidate the genomic footprints of recent positive selection within the YE, we conducted a genome-wide scan utilizing the iHS method. Given that the reliability of detected selection signals is contingent upon the statistical robustness of the calculated scores, we first characterized the distributional properties of the iHS values and their associated significance levels (Figure 4), The genome-wide distribution of iHS values approximated a standard normal distribution, exhibiting a mean centered near zero (μ = 1) and a standard deviation of approximately one (σ = 0). The fitted Gaussian curve (red line) demonstrated a high degree of congruence with the observed frequency histogram (Figure 4C,D). Furthermore, the quantile–quantile (Q-Q) plot (Figure 4E) indicated that the observed iHS values closely adhered to the theoretical normal distribution line. This corroborates the hypothesis that the vast majority of SNP loci represent a state of genomic neutrality. In contrast, the distribution of significance values exhibited a pronounced right-skewed pattern (skewness = 4.76) characterized by a long tail (Figure 4F). This distribution is quintessential for statistical significance metrics, where extreme values denote potential targets of selection. Leveraging the strict normality of the iHS distribution, SNPs falling at the extreme tails were identified as potential candidate loci indicative of selective sweeps (Figure 4G, Table S3). Specifically, employing a threshold of |iHS| > 2, we identified multiple candidate genes within these selection signal regions, including *PLPP4*, *GHRL*, *IFT122*, *CDK1*, *TP53*, *STAT3*, *MAPK3*, *AKT1*, *LOC106040672*, and *LOC125182034*, etc. Notably, this set includes several uncharacterized genes (LOC-prefix) that currently lack formal nomenclature in the National Center for Biotechnology Information database. Functionally, these genes are intimately associated with critical traits in the YE, including growth and development, lipid deposition, and disease resistance and stress resilience.

#### 3.3.2. Functional Annotation and Pathway Enrichment Analysis of Candidate Genes

**Figure 5:**
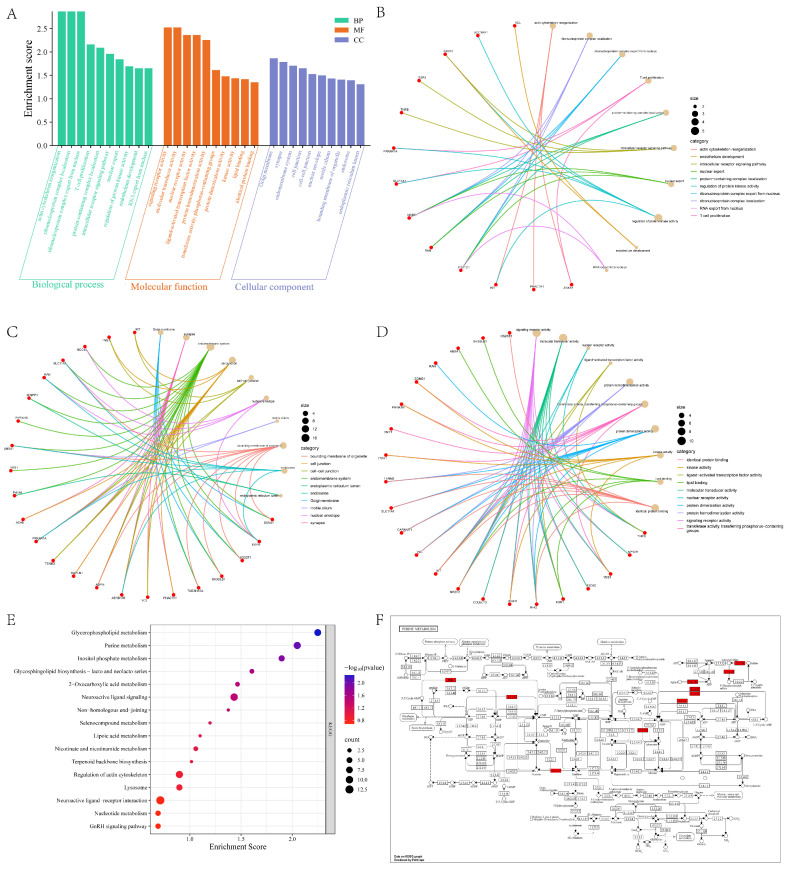
Functional annotation and enrichment analysis of candidate genes: (**A**) Histogram of GO enrichment analysis. The bar chart displays the enrichment scores for significant GO terms, categorized and color-coded as follows: Biological Process (BP, green), Molecular Function (MF, orange), and Cellular Component (CC, purple). (**B**–**D**) Chord diagrams illustrating associations between candidate genes and significant GO terms. The panels correspond to Biological Process, Molecular Function, and Cellular Component, respectively. Gene symbols are listed on the left arc and are connected via chords to their corresponding functional terms on the right arc. Genes of the same color represent the same pathway, while genes of different colors represent the opposite. (**E**) Bubble plot of KEGG pathway enrichment analysis. The x-axis represents the enrichment score, while the y-axis lists the pathway names. The size of each bubble is proportional to the number of enriched candidate genes, and the color gradient indicates the level of statistical significance. (**F**) Schematic of the purine metabolism pathway. Red boxes highlight the candidate genes identified within the potential selective sweep regions of the YE genome.

To delineate the biological functions of the putatively selected candidate genes in the YE, we performed GO and KEGG enrichment analyses (Figure 5). The GO analysis categorized candidate genes into Biological Process (BP), Cellular Component (CC), and Molecular Function (MF) classes (Figure 5A), with the top five most significantly enriched terms in each category summarized (Table 4). GO enrichment results within the BP category revealed that candidate genes were significantly enriched for terms related to cellular organization and immune response, such as ‘actin cytoskeleton organization’ and ‘T cell proliferation’. The chord diagram (Figure 5B) illustrates that genes such as *VCL*, *KIT*, and *PHACTR1* are intimately linked to these processes. In the MF category (Figure 5C), candidate genes were primarily enriched for ‘signaling receptor activity’ and ‘molecular transducer activity’. Key genes such as *ESR1*, *THRB*, and *NR2F2* were identified in these categories, suggesting that hormone regulation and signal transduction constitute important targets of selection. In the CC class (Figure 5D), significant enrichment was observed for ‘Golgi membrane’, ‘synapse’, and ‘endomembrane system’. Notably, genes such as *EGFR*, *SH3GLB1*, and *TENM2* were enriched within these cellular structures, indicating that cellular signaling and structural integrity represent potential adaptive directions. The KEGG pathway enrichment analysis (Figure 5E) further revealed significant enrichment of candidate genes in metabolic and signaling pathways, with the top ten most significantly enriched pathways detailed (Table 5). The most highly enriched pathways included ‘Glycerophospholipid metabolism’ (*p* < 0.01), ‘Purine metabolism’ (*p* < 0.01), and ‘Inositol phosphate metabolism’ (Figure S1). The enrichment of genes such as *PLA2G15*, *PLPP4* (in Glycerophospholipid metabolism), *ENPP3*, and *ADK* (in Purine metabolism) underscores the critical role of lipid and nucleotide metabolism in the adaptation of the YE. Specifically, the ‘Purine metabolism’ pathway demonstrates that the identified candidate genes (marked in red) play crucial roles within the metabolic network (Figure 5F). Furthermore, pathways related to environmental information processing, such as ‘Neuroactive ligand-receptor interaction,’ also showed enrichment, involving genes like *HTR4* and *GRIA2*. This may confer advantages for the population’s behavioral and physiological adaptation.

## 4. Discussion

### 4.1. Genomic Landscape of Single Nucleotide Variations

Whole-genome resequencing has established itself as a pivotal instrument for elucidating the genetic architecture of livestock populations and evaluating germplasm characteristics. In the present study, we identified an average of 4.43 million SNPs across the 39 chromosome pairs of the YE. In contrast, Wen et al. [20], in their genomic investigation into the origins of domestic geese, reported 24.89 million SNPs—a figure significantly exceeding the count observed in our study. This discrepancy is likely attributable to two primary factors. First, the relatively lower sequencing depth employed in this study (6.4× < 11.2×) may have constrained the detection of low-frequency variants. Second, substantial divergence exists between the two study populations regarding their genetic backgrounds and breeding histories.

Additionally, the transversion-to-transversion (Ti/Tv) ratio in geese aligns with the average levels observed in other goose breeds (2.46 > 2.40), yet remains within the typical range for waterfowl species. This reflects a conservative preference in mutation mechanisms, where transversions are less likely than transitions [21]. The genomic distribution of SNPs parallels findings from prior studies, characterized by a predominant accumulation of variants within intergenic and intronic regions [22]. This accumulation pattern in non-coding regions is consistent with the neutral theory of molecular evolution, which predicts a higher tolerance for mutations in sequences under relaxed functional constraints. Intriguingly, we observed a ratio of synonymous (33,677) to non-synonymous (11,735) SNPs of approximately 2.87. This ratio is significantly higher than that derived from Huoyan goose data (2.35) [23]. Such a discrepancy implies that the YE goose may have undergone stronger purifying selection, effectively purging deleterious non-synonymous mutations to preserve protein structural stability. Although rigorous quality control was applied, the potential for phasing uncertainty due to limited sequencing depth remains a limitation. Future studies with higher sequencing depth are therefore recommended to verify these observations.

### 4.2. Analysis of Genetic Structure in the Conservation Population

Further elucidation of the target population’s genetic structure is fundamental for assessing conservation efficacy and formulating effective breeding strategies. Our IBD distance and G-matrix analyses revealed a relatively tight and uniform genetic relationship within the YE (average IBD = 0.24), with no evidence of significant family stratification. This outcome suggests that the exsitu conservation farm has been successful in maintaining population size and implementing random mating protocols, thereby efficaciously mitigating severe genetic drift or subpopulation differentiation often associated with closed, small-population breeding. Intriguingly, individuals YE.5 and YE.9 exhibited an elevated IBD distance (0.31) relative to the rest of the cohort. This finding parallels observations by Ouyang et al. [24] in their studies of Chinese indigenous goose evolution, where certain individuals displayed evidence of admixture or distinct evolutionary pathways. This difference in genetic background may stem from the retention of unique ancestral haplotypes, which should be a key focus for prioritization and utilization in future selective breeding programs.

The rate of LD decay is a crucial metric reflecting population genetic diversity and recombination history. Our study showed that the YE experienced a rapid decay, with r^2^ dropping below 0.3 at approximately 50 kb. This decay rate is comparable to that reported for other Chinese indigenous goose breeds (*Anser cygnoides*) [25,26], suggesting that the YE breeding history involved frequent genetic recombination, thereby sustaining a high level of genetic diversity. Furthermore, considering the potential haplotype phasing uncertainties at 6× sequencing depth, we chose to use the PSMC method to avoid biases associated with haplotype-dependent methods. We recommend that future studies utilize high-depth sequencing data and employ these newer algorithms to decipher the recent population dynamics trajectories of geese. PSMC analysis of the Ne revealed two major historical bottleneck events. Specifically, during the LGM (15 to 21 thousand years ago), Ne declined sharply to its minimum; this finding corroborates the notion that habitat loss and resource scarcity due to global cooling were the primary constraints limiting the expansion of ancient avian populations [27]. Subsequently, the Ne entered a stabilization phase (plateau) approximately 10 thousand years ago. This timing coincides with the Neolithic period, when humans initiated the domestication of poultry globally. Compared to wild counterparts, domesticated geese benefited from the stability of human protection, buffering them against intense natural selection pressures [28]. This finding not only corroborates the shared historical background of goose (*Anser cygnoides*) domestication in China but also provides crucial genomic evidence for tracing the ancestral origins of the YE breed.

### 4.3. Genomic Signatures of Positive Selection and Their Biological Implications

In the present study, we leveraged whole-genome SNP data to conduct a genome-wide scan for selection signatures within the conservation population of YE, employing the iHS method. The iHS statistic offers a distinct advantage in detecting ongoing or recent positive selection signatures that have not yet reached fixation. Consequently, it is particularly efficacious in capturing the genomic “selection footprints” imprinted during the processes of domestication and artificial breeding [29]. By applying a defined screening threshold, we successfully identified a suite of candidate genes subject to potential selection, including *PLPP4*, *GHRL*, *IFT122*, *CDK1*, *TP53*, *STAT3*, *MAPK3*, and *AKT1*. Functional analysis revealed that these genes are predominantly enriched in biological processes encompassing growth and development, lipid metabolism, and immune response. These findings align closely with the distinguishing germplasm characteristics of the YE as a superior meat-type breed, namely, its rapid growth rate, large body size, and robust stress resilience.

First, growth traits constitute a primary target of intense artificial selection during the domestication of poultry. Within the identified genomic regions harboring strong selection signatures, we detected multiple pivotal genes intimately associated with the growth axis and cellular proliferation, notably *GHRL*, *AKT1*, and *MAPK3*. Specifically, the *GHRL* encodes the ghrelin–obestatin preproprotein, a precursor to ghrelin. As the endogenous ligand for the growth hormone secretagogue receptor (*GHS-R*), it plays a central orchestration role in regulating feed intake behavior, maintaining energy homeostasis, and stimulating growth hormone secretion. Previous investigations by Li et al. [30] into the growth patterns of dwarf chickens demonstrated that GHRL functions as an appetite modulator, influencing nutrient absorption by regulating feed intake. Consequently, the enrichment of GHRL variants in the YE genome may be linked to the breed’s enhanced foraging capacity and somatic development. Furthermore, *AKT1* and *MAPK3* serve as canonical components of the PI3K-Akt and *MAPK* signaling pathways, respectively. These pathways are integral to cellular proliferation, differentiation, and the process of skeletal muscle myogenesis [31,32]. The identification of these key signaling members suggests that genomic regions governing muscle development in the YE have undergone significant adaptive evolution during long-term selective breeding. This evolutionary process has likely established the genetic architecture underpinning the breed’s superior meat production performance.

Second, selection signatures associated with lipid metabolism illuminate the potential genetic mechanisms underlying the meat quality traits of the Yan goose. The identified candidate genes, including *PLPP4*, *SAMD8*, and *LPIN1*, are integral to pathways such as glycerophospholipid metabolism and lipid synthesis, playing pivotal roles in these metabolic processes [33,34]. Waterfowl, particularly geese, possess a distinctive capacity for adipose deposition (exemplified by hepatic lipid metabolism). Consequently, the positive selection acting on these lipid-related genes is likely associated with intramuscular fat deposition or body fat distribution patterns, thereby influencing meat flavor and texture. Synthesizing these findings with the KEGG enrichment results, the significant enrichment of ‘Purine metabolism’ and ‘Inositol phosphate metabolism’ pathways further implies a distinct metabolic plasticity in energy homeostasis and substrate turnover. This adaptation likely underpins the YE’s tolerance to coarse feed and its high feed conversion efficiency.

Finally, this study substantiated the environmental adaptability and disease resistance of the YE at the genomic level. The identified *TP53* and *STAT3* genes serve as critical mediators of cellular stress responses and immune regulation [35]. As elucidated by Baliakas et al. [36] in their investigation of the *TP53* tumor suppressor, *p53* functions as a “master switch” bridging cellular stress and appropriate cellular or multicellular responses. Similarly, *STAT3* acts as a central transcription factor within the JAK-STAT signaling pathway, directly facilitating cytokine signal transduction and immune cell activation [37]. The positive selection acting on these immunity-related genes likely signifies the evolution of adaptive genetic mechanisms conferred to the YE. These mechanisms have evolved to counteract pathogen invasion and cope with environmental stressors throughout their long natural history and domestication.

### 4.4. Limitations of the Study

While this study provides the first comprehensive genomic characterization of the Yan goose, we acknowledge certain limitations. First, the sample size (N = 15) is relatively modest, which is a common constraint in studies involving specific conserved indigenous breeds. Although the iHS method serves as a robust statistic for detecting signatures of recent positive selection, its application to smaller datasets may introduce statistical noise or reduce the power to detect low-frequency variants. To mitigate these potential biases, we implemented stringent quality control measures and conservative detection thresholds; nevertheless, the possibility of false positives cannot be entirely ruled out. Second, our sampling strategy was restricted to a single ex situ conservation population. While this population captures the core germplasm of the breed, future investigations should aim to incorporate larger cohorts from diverse agroecological zones and conduct comparative genomic analyses with other domestic goose breeds and wild *Anser cygnoides* (*Anser cygnoides*). Such endeavors will be instrumental in further validating the specificity of the identified selection signatures and refining the evolutionary trajectory of the breed.

## 5. Conclusions

In this study, we employed whole-genome resequencing technology to provide the first systematic elucidation of the genomic variation profiles and evolutionary history characterizing the conserved YE breed. Our findings substantiate the rich genetic diversity and distinct evolutionary trajectory of this breed, while clearly reconstructing the historical demographic sculpting exerted by climatic fluctuations during the Quaternary glaciation. Crucially, our analyses successfully pinpointed key candidate genes intimately associated with growth and development (*GHRL*, *AKT1*, and *MAPK3*), lipid metabolism (*PLPP4*, *SAMD8*, and *LPIN1*), and immune adaptation (*TP53*, *STAT3*). These discoveries decipher the molecular mechanisms underlying the YE’s distinguishing germplasm attributes, specifically its large body size, superior meat quality, and robust stress resilience at the genomic level. Furthermore, this work establishes a robust foundation for future endeavors in molecular marker-assisted selection and genomic breeding programs targeting economically important traits in domestic geese.

## Figures and Tables

**Table 1:** Statistical summary of identified SNPs and variant parameters.

Item	SNP	Ti	Tv	Ti/Tv (%)	He	Ho
YE.1	4,249,848	3,026,441	1,223,407	2.47	2,033,435	2,216,413
YE.2	4,516,551	3,212,443	1,304,108	2.46	2,265,592	2,250,959
YE.3	4,193,357	2,986,922	1,206,435	2.47	1,925,688	2,267,669
YE.4	4,521,725	3,214,828	1,306,897	2.45	2,229,089	2,292,636
YE.5	4,868,607	3,464,303	1,404,304	2.46	2,517,574	2,351,033
YE.6	4,069,187	2,896,573	1,172,614	2.47	1,825,696	2,243,491
YE.7	4,116,905	2,934,630	1,182,275	2.48	1,952,927	2,163,978
YE.8	4,427,089	3,153,851	1,273,238	2.47	2,388,968	2,038,121
YE.9	4,634,450	3,308,984	1,325,466	2.49	2,358,672	2,275,778
YE.10	4,442,781	3,168,351	1,274,430	2.48	2,221,703	2,221,078
YE.11	4,264,640	3,033,130	1,231,510	2.46	1,969,277	2,295,363
YE.12	4,725,693	3,372,046	1,353,647	2.49	2,609,066	2,116,627
YE.13	4,518,224	3,215,974	1,302,250	2.46	2,106,137	2,412,087
YE.14	4,655,819	3,314,398	1,341,421	2.47	2,428,996	2,226,823
YE.15	4,231,364	3,018,327	1,213,037	2.48	1,952,839	2,278,525
Average	4,429,082	3,154,746	1,274,335	2.47	2,185,710	2,243,372

Note: Ti, transition variants (purine-to-purine or pyrimidine-to-pyrimidine substitutions); Tv, transversion variants (purine-to-pyrimidine or pyrimidine-to-purine substitutions); Ti/Tv, transition-to-transversion ratio; He, heterozygous SNP count (number of sites where two different alleles are present); Ho, homozygous SNP count (number of sites where both alleles are the same).

**Table 2:** Statistical summary of intraspecies SNP locus annotation.

Item	5′ UTR	AS	Syn	Non-Syn	Int	3′ UTR	Up/Down	Inter
YE.1	57,391	125	30,613	10,615	1,607,168	61,687	9424	1,084,836
YE.2	60,590	112	33,166	11,951	1,684,661	67,325	9837	1,127,935
YE.3	58,248	134	32,744	11,700	1,662,763	63,397	9474	1,089,767
YE.4	59,597	118	31,710	11,206	1,671,684	64,494	9791	1,116,326
YE.5	77,569	177	41,578	14,321	2,171,599	84,188	12,713	1,447,039
YE.6	54,333	121	30,105	10,192	1,547,760	60,042	9140	1,023,064
YE.7	59,112	149	31,765	10,628	1,632,537	64,367	9600	1,085,012
YE.8	67,792	150	36,184	12,314	1,901,127	73,891	10,832	1,276,499
YE.9	77,505	154	41,539	14,524	2,131,764	84,826	12,549	1,438,706
YE.10	61,007	124	32,784	11,658	1,666,449	65,284	9866	1,128,659
YE.11	58,464	123	31,430	10,475	1,623,541	63,048	9434	1,079,978
YE.12	66,266	152	34,974	12,446	1,819,685	72,023	10,751	1,223,139
YE.13	62,411	122	32,830	11,287	1,708,629	67,977	9937	1,167,553
YE.14	62,633	140	33,327	11,840	1,735,879	67,548	10,102	1,160,186
YE.15	57,282	146	30,406	10,878	1,565,366	61,424	9139	1,057,823
Average	62,680	136	33,677	11,735	1,742,040	68,101	10,172	1,167,101

Note: 5′ UTR, variants located in the 5′ untranslated region; AS, variants located in alternative splicing sites; Syn, synonymous variants (no amino acid change); Non-syn, non-synonymous variants (causing amino acid change); Int, variants located in intronic regions; 3′ UTR, variants located in the 3′ untranslated region; Up/Down, variants located in upstream or downstream regions (proximal to genes); Inter, variants located in intergenic regions.

**Table 3:** Statistical summary of IBD genetic distances.

Item	YE.1	YE.2	YE.3	YE.4	YE.5	YE.6	YE.7	YE.8	YE.9	YE.10	YE.11	YE.12	YE.13	YE.14	YE.15
YE.1		0.22	0.23	0.23	0.31	0.23	0.24	0.26	0.31	0.23	0.23	0.24	0.25	0.23	0.16
YE.2	0.22		0.23	0.23	0.31	0.22	0.24	0.26	0.31	0.23	0.23	0.24	0.25	0.22	0.23
YE.3	0.23	0.23		0.24	0.31	0.23	0.24	0.26	0.31	0.24	0.24	0.22	0.25	0.24	0.24
YE.4	0.23	0.23	0.24		0.31	0.24	0.24	0.27	0.31	0.24	0.22	0.23	0.23	0.22	0.23
YE.5	0.31	0.31	0.31	0.31		0.31	0.31	0.30	0.25	0.31	0.31	0.31	0.31	0.31	0.31
YE.6	0.23	0.22	0.23	0.24	0.31		0.20	0.26	0.31	0.22	0.24	0.23	0.25	0.20	0.24
YE.7	0.24	0.24	0.24	0.24	0.31	0.20		0.26	0.31	0.22	0.25	0.23	0.25	0.23	0.24
YE.8	0.26	0.26	0.26	0.27	0.30	0.26	0.26		0.30	0.26	0.25	0.23	0.24	0.25	0.23
YE.9	0.31	0.31	0.31	0.31	0.25	0.31	0.31	0.30		0.31	0.31	0.31	0.31	0.31	0.31
YE.10	0.23	0.23	0.24	0.24	0.31	0.22	0.22	0.26	0.31		0.23	0.24	0.25	0.22	0.23
YE.11	0.23	0.23	0.24	0.22	0.31	0.24	0.25	0.25	0.31	0.23		0.22	0.25	0.24	0.23
YE.12	0.24	0.24	0.22	0.23	0.31	0.23	0.23	0.23	0.31	0.24	0.22		0.23	0.23	0.23
YE.13	0.25	0.25	0.25	0.23	0.31	0.25	0.25	0.24	0.31	0.25	0.25	0.23		0.23	0.25
YE.14	0.23	0.22	0.24	0.22	0.31	0.20	0.23	0.25	0.31	0.22	0.24	0.23	0.23		0.24
YE.15	0.16	0.23	0.24	0.23	0.31	0.24	0.24	0.23	0.31	0.23	0.23	0.23	0.25	0.24	

Note: YE.1–15, identifiers for the 15 Yan goose individuals; values in the matrix represent the IBD (Identity-by-Descent) genetic distances between pairs of individuals.

**Table 4:** GO enrichment results for genes located within high-frequency ROH regions.

Item	ID	Terms	N	Gene Name	*p*
BP	GO:0038023	Signaling receptor activity	10	*THRB*/*NPY2R*/*YES1*/*FZD10*/*ESR1*/*RHO*/*EGFR*/*COLEC12*/*NR2F2*/*KIT*	2.98 × 10^−3^
	GO:0060089	Molecular transducer activity	10	*THRB*/*NPY2R*/*YES1*/*FZD10*/*ESR1*/*RHO*/*EGFR*/*COLEC12*/*NR2F2*/*KIT*	2.98 × 10^−3^
	GO:0046983	Protein dimerization activity	8	*VCL/RHO*/*CARNMT1/RAN*/*SLC11A1*/*TENM2*/*NR2F2*/*KIT*	3.33 × 10^−2^
	GO:0042802	Identical protein binding	8	*VCL*/*RHO*/*CARNMT1*/*HS2ST1*/*SLC11A1*/*TENM2*/*NR2F2*/*KIT*	4.46 × 10^−2^
	GO:0042803	Protein homodimerization activity	7	*VCL*/*RHO*/*CARNMT1*/*SLC11A1*/*TENM2*/*NR2F2*/*KIT*	5.53 × 10^−3^
CC	GO:0000139	Golgi membrane	5	*SGMS1*/*EGFR*/*HS2ST1*/*SH3GLB1*/*TMEM167A*	1.36 × 10^−2^
	GO:0045202	Synapse	8	*PHACTR1*/*VCL*/*ABHD17B*/*AMPH*/*HAPLN1*/*TENM2*/*PRKAR1A*/*ACHE*	1.63 × 10^−2^
	GO:0012505	Endomembrane system	17	*P4HA1*/*YES1*/*ANXA1*/*PIP5K1B*/*MINPP1*/*SGMS1*/*EGFR*/*HS2ST1*/*SH3GLB1*/*TMEM167A*/*ABHD17B*/*AMPH*/*RAN*/*SLC11A1*/*TENM2*/*PRKAR1A*/*NOC4L*	1.97 × 10^−2^
	GO:0030054	Cell junction	10	*PHACTR1*/*VCL*/*ABHD17B*/*AMPH*/*TNS1*/*HAPLN1*/*TENM2*/*PRKAR1A*/*KIT*/*ACHE*	2.23 × 10^−2^
	GO:0005911	Cell-cell junction	4	*VCL*/*TNS1*/*TENM2*/*KIT*	2.95 × 10^−2^
MF	GO:0038023	Signaling receptor activity	10	*THRB*/*NPY2R*/*YES1*/*FZD10*/*ESR1*/*RHO*/*EGFR*/*COLEC12*/*NR2F2*/*KIT*	2.90 × 10^−3^
	GO:0060089	Molecular transducer activity	10	*THRB*/*NPY2R*/*YES1*/*FZD10*/*ESR1*/*RHO*/*EGFR*/*COLEC12*/*NR2F2*/*KIT*	2.90 × 10^−3^
	GO:0004879	Nuclear receptor activity	3	*THRB*/*ESR1*/*NR2F2*	4.30 × 10^−3^
	GO:0098531	Ligand-activated transcription factor activity	3	*THRB*/*ESR1*/*NR2F2*	4.30 × 10^−3^
	GO:0042803	Protein homodimerization activity	7	*VCL*/*RHO*/*CARNMT1*/*SLC11A1*/*TENM2*/*NR2F2*/*KIT*	5.50 × 10^−3^

Note: GO category refers to the main category of Gene Ontology terms; BP (Biological Process), CC (Cellular Component), and MF (Molecular Function) are the three main classifications of GO terms. ‘Terms’ refers to the specific GO functional description; ‘N’ refers to the number of candidate genes enriched in this GO term; ‘Gene name’ lists the genes associated with the GO term; *p* refers to the significance of enrichment (*p*-value).

**Table 5:** KEGG pathway enrichment results for genes located within high-frequency ROH regions.

ID	Pathway	N	Genes Name	*p*
gga00564	Glycerophospholipid metabolism	8	*PLA2G15*/*PLPP4*/*PLA2G4B*/*SAMD8*/*SGMS1*/*ACHE*/*PLA2G12B*/*LPIN1*	5.60 × 10^−3^
gga00230	Purine metabolism	9	*ENPP3*/*PDE1A*/*PDE10A*/*PDE1C*/*PDE7B*/*FHIT*/*ADK*/*PAPSS2*/*GDA*	8.90 × 10^−3^
gga00562	Inositol phosphate metabolism	6	*PTEN*/*ITPK1*/*PIP5K1B*/*PIKFYVE*/*MINPP1*/*INPP5A*	1.26 × 10^−2^
gga00601	Glycosphingolipid biosynthesis lacto and neolacto series	3	*A4GALT*/*B4GALT1*/*B3GNT4*	2.47 × 10^−2^
gga01210	2-Oxocarboxylic acid metabolism	3	*OGDHL*/*IDH1*/*BCKDHB*	3.42 × 10^−2^
gga04082	Neuroactive ligand signaling	10	*SLC1A1*/*GRIA2*/*GRIA4*/*GNAL*/*GABBR2*/*HTR4*/*GLRA1*/*GLRB*/*ACHE*/*GNG10*	3.69 × 10^−2^
gga03450	Non-homologous end-joining	2	*DNTT*/*XRCC4*	4.21 × 10^−2^
gga00450	Selenocompound metabolism	2	*KYAT3*/*PAPSS2*	6.35 × 10^−2^
gga00785	Lipoic acid metabolism	2	*OGDHL*/*BCKDHB*	7.94 × 10^−2^
gga00760	Nicotinate and nicotinamide metabolism	3	*ENPP3*/*NUDT13*/*NMRK1*	8.75 × 10^−2^

Note: ID, the identifier of the pathway in the Kyoto Encyclopedia of Genes and Genomes database; Pathway, the name of the metabolic or signaling pathway; N, number of candidate genes enriched in the pathway; Genes, list of candidate genes associated with the pathway; *p*, statistical significance of the enrichment (*p*-value).

## Data Availability

All data generated or analyzed during this study are included in this published paper.

## Supplementary Materials

The following supporting information can be downloaded at: https://www.mdpi.com/article/10.3390/ani16020194/s1, Table S1: Statistical Summary of Sequencing Data Quality for the Yan Goose; Table S2: Statistical Summary of Sequencing Read Alignment for the Yan Goose; Table S3: Screening of candidate genes for geese based on the integrated haplotype score method; Figure S1: KEGG pathway enrichment analysis diagram. The red boxes highlight candidate genes identified within potential selection regions of the Yan goose genome.
