# Supplementary material for: Unveiling the Genomic Landscape of Yan Goose (Anser cygnoides): Insights into Population History and Selection Signatures for Growth and Adaptation
Source: Animals (Basel). 2026 Jan 8;16(2):194. doi: 10.3390/ani16020194 (PMC12838169; doi:10.3390/ani16020194)
Supplement: Supplementary file 1 [file animals-16-00194-s001.zip › Supplementary materials -Table S2.pdf]

Table S2. Statistical Summary of Sequencing Read Alignment for the Yan Goose

Item	Total reads	Mapped reads	Alignment Rate (%)	Mean Depth (×)
YE.1	42,176,822	41,466,754	98.32	5.81
YE.2	49,234,360	48,384,430	98.27	6.73
YE.3	41,218,932	40,511,985	98.28	5.67
YE.4	51,537,852	50,718,130	98.41	7.05
YE.5	52,086,386	51,214,793	98.33	7.12
YE.6	40,031,376	39,301,785	98.18	5.53
YE.7	38,950,912	38,277,282	98.27	5.39
YE.8	41,853,446	41,129,645	98.27	5.76
YE.9	45,961,698	45,238,692	98.43	6.36
YE.10	47,005,324	46,174,283	98.23	6.47
YE.11	42,756,966	42,102,131	98.47	5.88
YE.12	57,633,768	56,708,791	98.4	7.94
YE.13	53,905,846	52,973,842	98.27	7.36
YE.14	53,659,316	52,693,096	98.2	7.32
YE.15	44,103,872	43,322,586	98.23	6.06
Average	46,807,791	46,014,548	98.30	6.43

Note: Total Reads, the total number of sequencing reads generated. A single pair-end fragment is counted as two individual reads; Mapped Reads, the count of reads successfully aligned (mapped) to the reference genome; Alignment Rate, the percentage of total reads successfully aligned to the reference genome; Mean Depth, the mean number of times each base in the reference genome was sequenced (coverage).
