# Supplementary material for: Unveiling the Genomic Landscape of Yan Goose (Anser cygnoides): Insights into Population History and Selection Signatures for Growth and Adaptation
Source: Animals (Basel). 2026 Jan 8;16(2):194. doi: 10.3390/ani16020194 (PMC12838169; doi:10.3390/ani16020194)
Supplement: Supplementary file 1 [file animals-16-00194-s001.zip › Supplementary materials -Table S1.pdf]

Table S1. Statistical Summary of Sequencing Data Quality for the Yan Goose

Item	Raw data	Raw reads	Clean data	Clean reads	Rate (%)	Q20	Q30	GC
YE.1	6.39	21,284,577	6.33	21,088,411	99.08	97.56	92.03	43.21
YE.2	7.46	24,852,081	7.39	24,617,180	99.05	97.57	92.08	43.03
YE.3	6.24	20,805,099	6.18	20,609,466	99.06	97.87	93.03	43.08
YE.4	7.80	26,005,495	7.73	25,768,926	99.09	97.58	92.32	42.82
YE.5	7.89	26,301,107	7.81	26,043,193	99.02	98.06	93.81	43.17
YE.6	6.06	20,199,586	6.00	20,015,688	99.09	97.46	91.64	43.08
YE.7	5.90	19,652,080	5.84	19,475,456	99.10	97.85	93.00	43.45
YE.8	6.34	21,132,640	6.28	20,926,723	99.03	97.72	92.44	43.28
YE.9	6.96	23,215,564	6.89	22,980,849	98.99	97.54	92.10	44.07
YE.10	7.11	23,715,033	7.05	23,502,662	99.10	97.31	90.11	44.03
YE.11	6.47	21,551,528	6.41	21,378,483	99.20	97.31	90.23	42.80
YE.12	8.73	29,097,067	8.65	28,816,884	99.04	96.50	88.23	44.20
YE.13	8.16	27,197,952	8.09	26,952,923	99.10	96.42	88.10	43.24
YE.14	8.12	27,066,821	8.05	26,829,658	99.12	97.26	90.07	43.56
YE.15	6.68	22,253,788	6.62	22,051,936	99.09	95.96	86.51	43.82
Average	7.08	23,622,027	7.02	23,403,895	99.07	97.33	92.03	43.38

Note: Raw Data, total sequencing data generated (measured in Gigabases, Gb); Raw Reads, total number of raw sequencing reads; Clean Data, high-quality data retained after filtering (Gb); Clean Reads, number of high-quality reads retained; Rate, effective utilization rate (percentage of clean data relative to raw data); Q20, percentage of bases with a Phred quality score > 20 (sequencing error rate < 1%); Q30, percentage of bases with a Phred quality score > 30 (sequencing error rate < 0.1%); GC, Guanine-Cytosine content percentage.
