# Supplementary material for: Unveiling the Genomic Landscape of Yan Goose (Anser cygnoides): Insights into Population History and Selection Signatures for Growth and Adaptation
Source: Animals (Basel). 2026 Jan 8;16(2):194. doi: 10.3390/ani16020194 (PMC12838169; doi:10.3390/ani16020194)
Supplement: Supplementary file 1 [file animals-16-00194-s001.zip › Supplementary materials -Figure S1.pdf]

Figure S1. KEGG pathway enrichment analysis diagram. The red boxes highlight candidate genes identified within potential selection regions of the Yan goose genome.

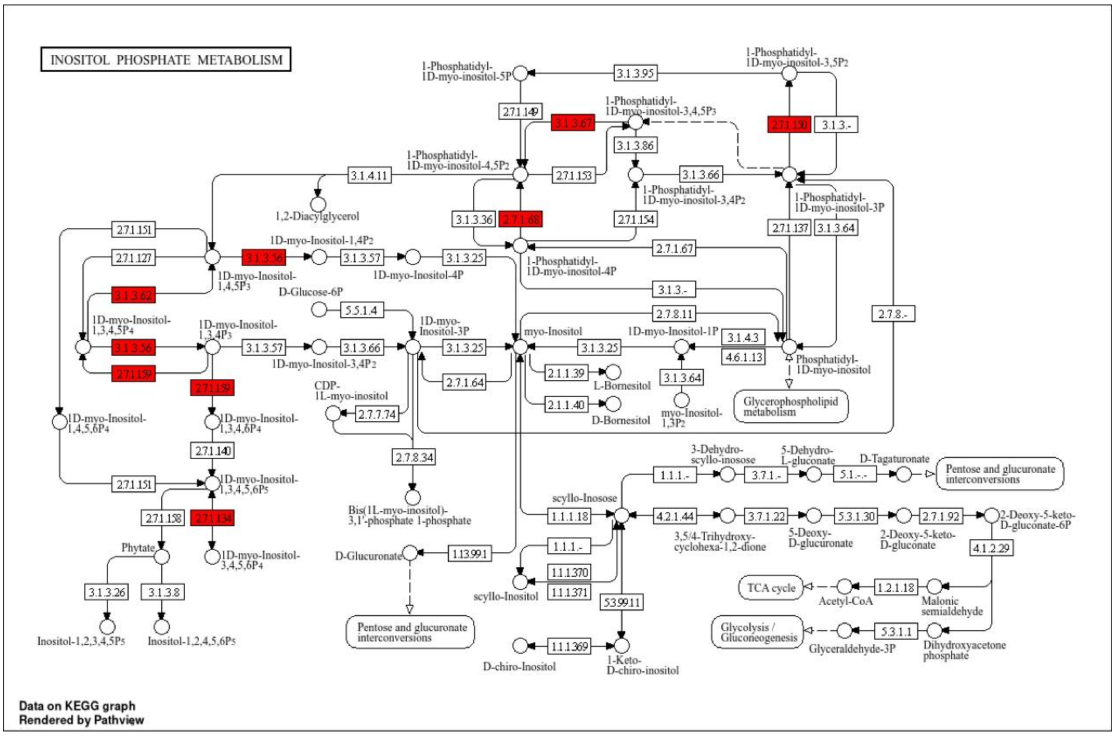

Inositol phosphate metabolism (gga00562) pathview

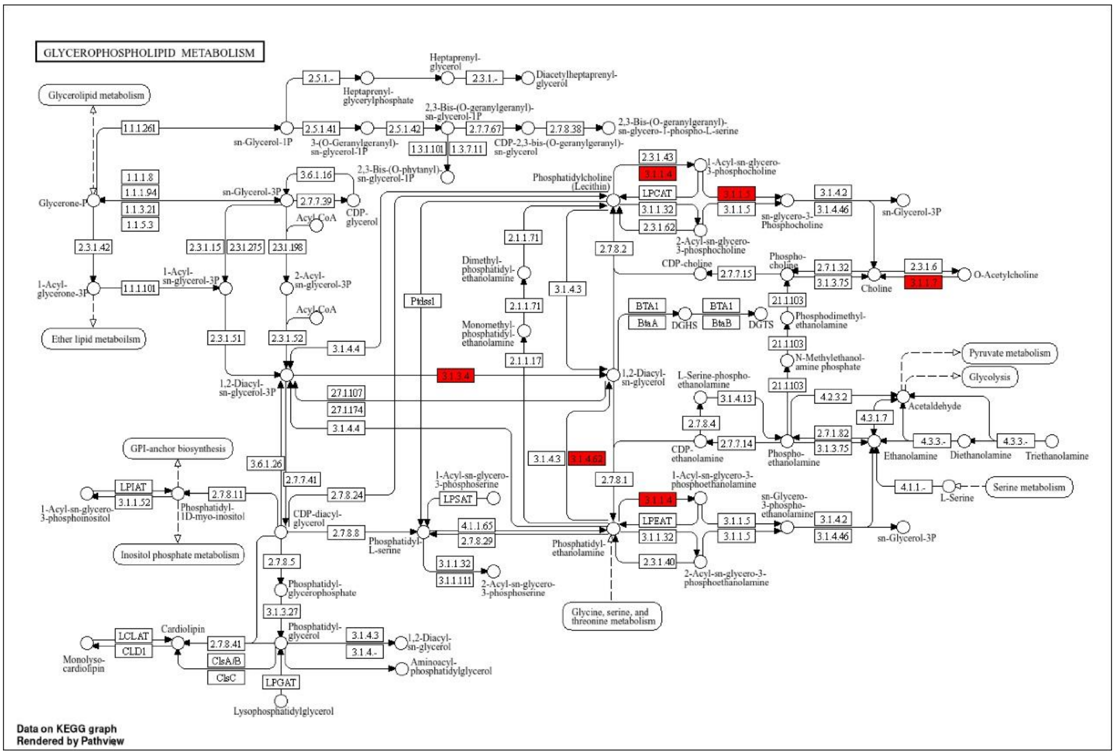

Glycerophospholipid metabolism (gga00564) pathview

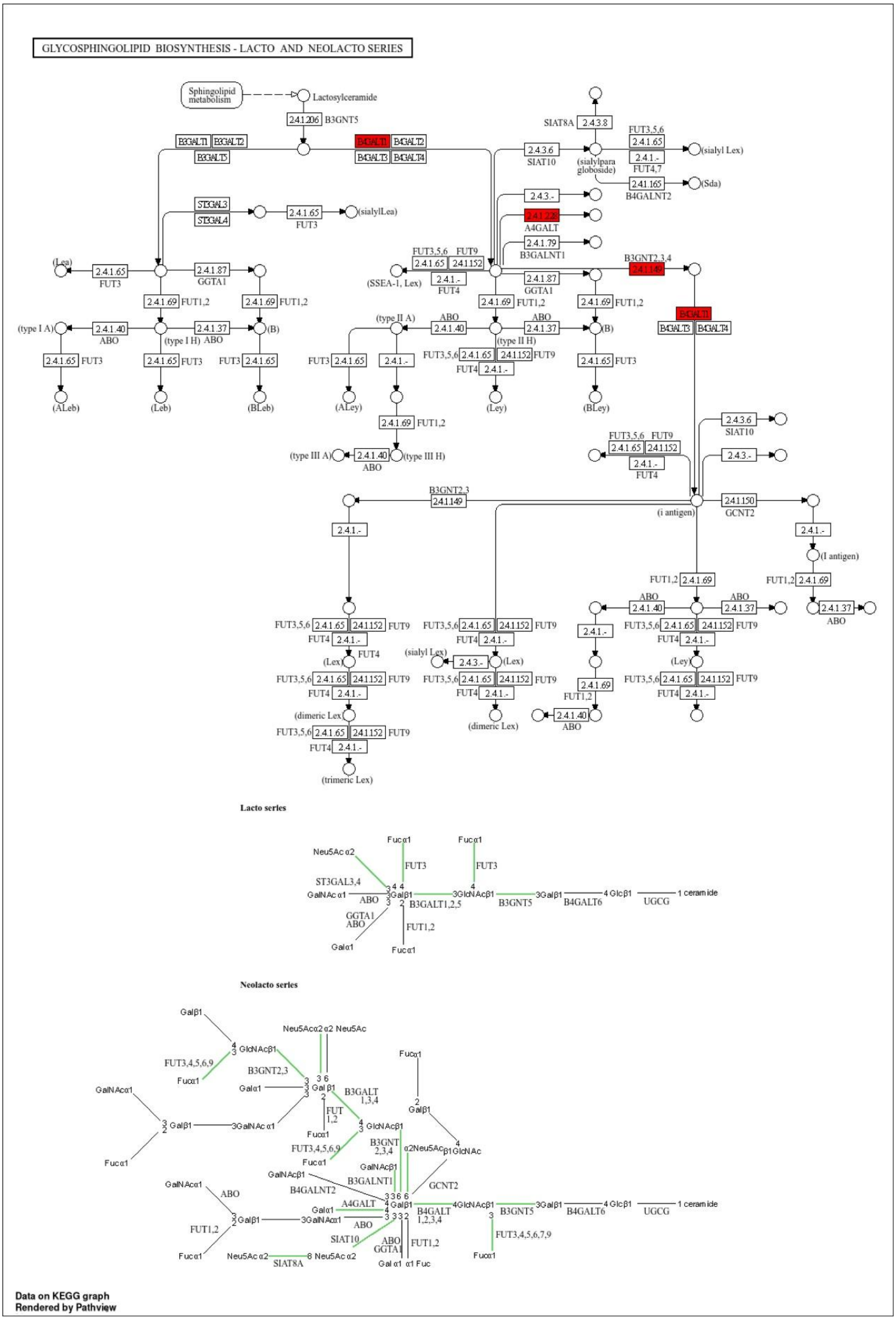

Glycosphingolipid biosynthesis lacto and neolacto series (gga00601) pathview

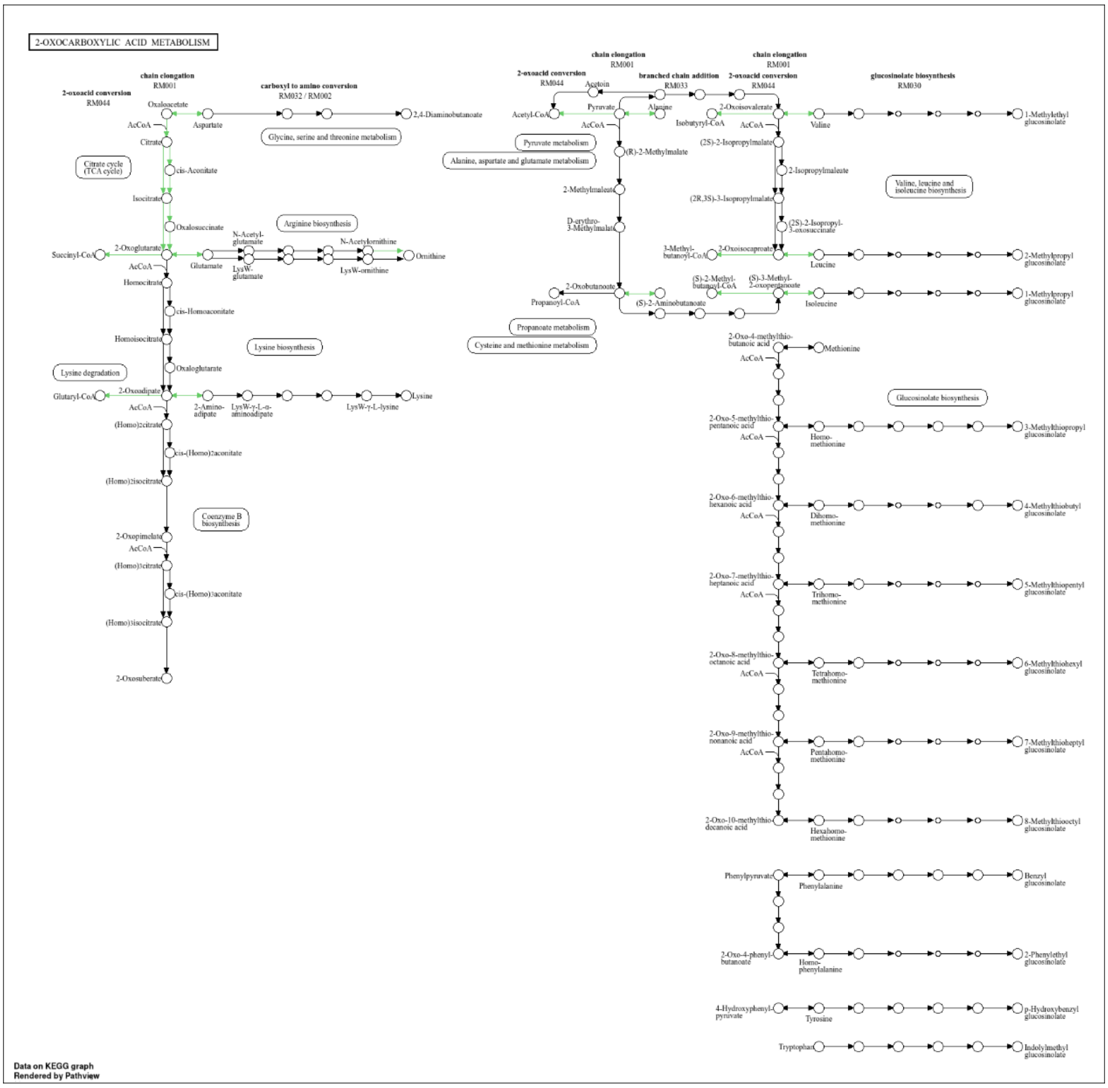

2-Oxocarboxylic acid metabolism (gga01210) pathview

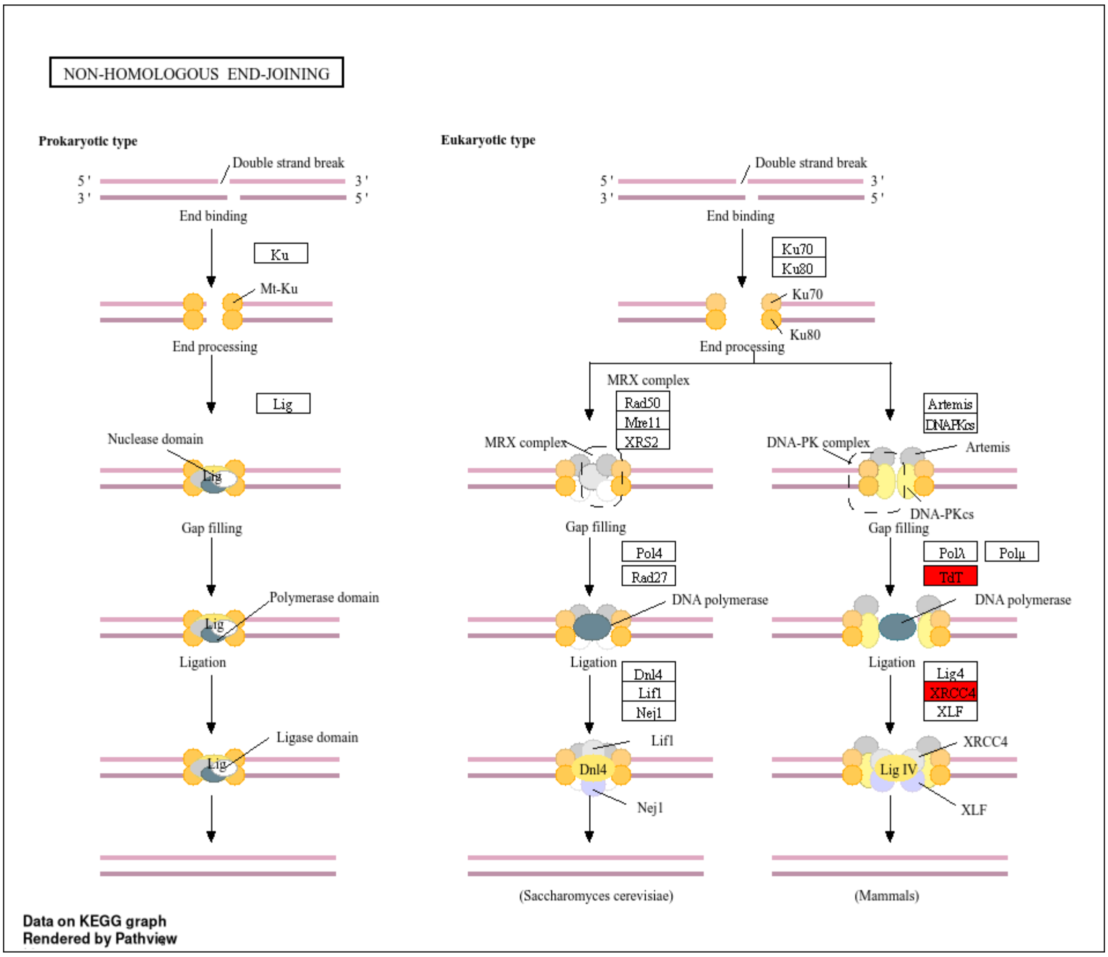

Non-homologous end-joining (gga03450) pathview

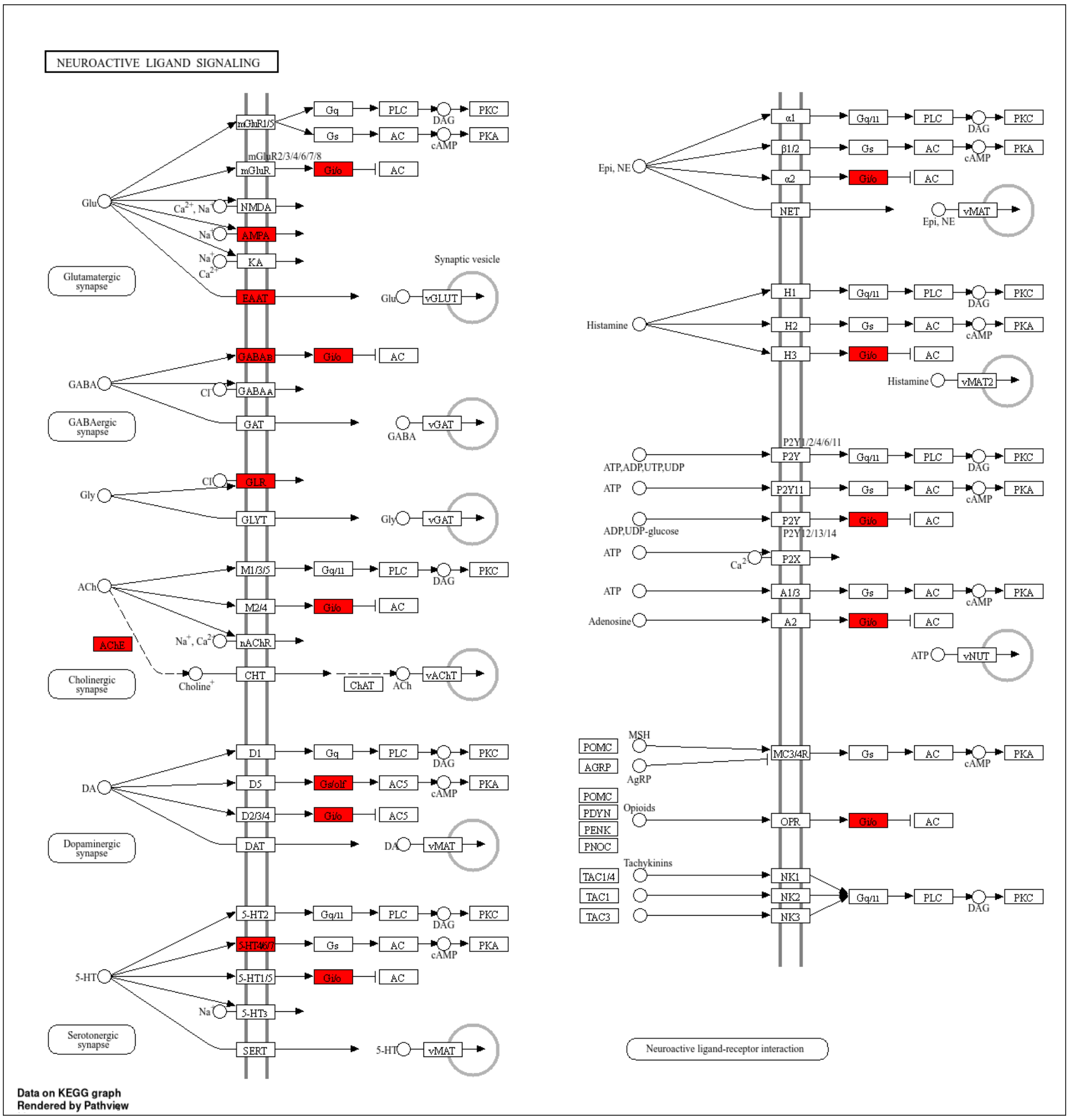

Neuroactive ligand signaling (gga04082) pathview
